# On Maternal Syphilis, Including the Presence and Recognition of Syphilitic Pelvic Disease in Women

**Published:** 1898-12

**Authors:** 


**On Maternal Syphilis, including the Presence and Recognition of Syphilitic Pelvic Disease in Women.** By JOHN A. SHAW-MACKENZIE, M.D. Pp. xi., 223. London: J. & A. Churchill. 1898.

Not only does this contain an account of the usual clinical manifestations of syphilis in women, but it is an able disquisition on some of the more debatable points of the subject. The author is an original thinker, and puts many questions in a new light. He is inclined to regard syphilis as a more potent factor in many pelvic diseases in women than is generally recognised; and thinks "that the maternal origin of syphilis in the offspring, in consequence of maternal acquired or inherited disease, is more probable, in doubtful cases, than the paternal origin, and 'syphilis by conception in the mother,'" as usually accepted. His views are well supported by argument and illustrative cases.

